# Preoperative Axillary Ultrasound versus Sentinel Lymph Node Biopsy in Patients with Early Breast Cancer

**DOI:** 10.3390/medicina56030127

**Published:** 2020-03-13

**Authors:** Dalia Rukanskienė, Vincentas Veikutis, Eglė Jonaitienė, Milda Basevičiūtė, Domantas Kunigiškis, Renata Paukštaitienė, Daiva Čepulienė, Lina Poškienė, Algirdas Boguševičius

**Affiliations:** 1 Department of Radiology, Faculty of Medicine, Medical Academy, Lithuanian University of Health Sciences, LT-50161 Kaunas, Lithuania; drebule@hotmail.com; 2 Institute of Cardiology, Faculty of Medicine, Medical Academy, Lithuanian University of Health Sciences, LT-50162 Kaunas, Lithuania; vincentas.veikutis@lsmuni.lt; 3 Faculty of Medicine, Medical Academy, Lithuanian University of Health Sciences, LT-44307 Kaunas, Lithuania; mbasev@gmail.com (M.B.); domantas.ku@gmail.com (D.K.); 4 Department of Physics, Mathematics and Biophysics, Faculty of Medicine, Medical Academy, Lithuanian University of Health Sciences, LT-44307 Kaunas, Lithuania; renata.paukstaitiene@gmail.com; 5 Department of Surgery, Faculty of Medicine, Medical Academy, Lithuanian University of Health Sciences, LT-50161 Kaunas, Lithuania; daiva.cepuliene@yahoo.com (D.Č.); algirdas.bogusevicius@kaunoklinikos.lt (A.B.); 6 Department of Pathological Anatomy, Faculty of Medicine, Medical Academy, Lithuanian University of Health Sciences, LT-50161 Kaunas, Lithuania; lina_poskiene@yahoo.com

**Keywords:** breast cancer, axillary lymph nodes, ultrasound, lymphadenectomy, sentinel lymph node biopsy

## Abstract

*Background and objectives:* With improved diagnostic means of early breast cancer, the percentage of cases with metastasis in axillary lymph nodes has decreased from 50–75% to 15–30%. Lymphadenectomy and sentinel lymph node biopsy are not treatment procedures, as they aim at axillary nodal staging in breast cancer. Being surgical interventions, they can lead to various complications. Therefore, recently much attention has been paid to the identification of non-invasive methods for axillary nodal staging. In many countries, ultrasound is a first-line method to evaluate axillary lymph node status. The aim of this study was to evaluate the prognostic value of ultrasound in detecting intact axillary lymph nodes and to assess the accuracy of ultrasound in detecting a heavy nodal disease burden. The additional objective was to evaluate patients’ and tumor characteristics leading to false-negative results. *Materials and Methods:* A total of 227 women with newly diagnosed pT1 breast cancer were included to this prospective study conducted at the Breast Surgery Unit, Clinic of Surgery, Hospital of Lithuanian University of Health Sciences Kauno Klinikos, between May 1, 2016, and May 31, 2018. All patients underwent preoperative axillary ultrasound examination. Ultrasound data were compared with the results of histological examination. The accuracy and true-negative rate of ultrasound were calculated. The reasons of false-negative results were analyzed. *Results:* Of the 189 patients who had normally appearing axillary lymph nodes on preoperative ultrasound (PAUS-negative), 173 (91.5%) patients were also confirmed to have intact axillary lymph nodes (node-negative) by histological examination after surgery. The accuracy and the negative predictive value of ultrasound examination were 84.1% and 91.5%, respectively. In ≥3 node-positive cases, the accuracy and the negative predictive value increased to 88.7% and 98.3%, respectively. In total, false-negative results were found in 8.5% of the cases (n = 16); in the PAUS-negative group, false-negative results were recorded only in 1.6% of the cases (n = 3). The results of PAUS and pathological examination differed significantly between patients without and with lymphovascular invasion (LV0 vs. LV1, *p* < 0.001) as well as those showing no human epidermal growth factor receptor 2 (HER2) expression and patients with weakly or strongly expressed HER2 (HER2(0) vs. HER2(1), *p* = 0.024). Paired comparisons revealed that the true-negative rate was significantly different between the LV0 and LV1 groups (91% vs. 66.7%, *p* < 0.05), and the false-negative rate was statistically significant different between the HER2(0) and HER2(1) groups (10.5% vs. 1.2%, *p* < 0.05). Evaluation of other characteristics showed both the groups to be homogenous. *Conclusions:* Negative axillary ultrasound excluded axillary metastatic disease in 91.5% of the patients. PAUS had an accuracy of 88.7% in detecting a heavy nodal disease burden. With the absence of lymphovascular invasion (LV0), we can rely on PAUS examination that axillary lymph nodes are intact (PAUS-negative), and this patients’ group could avoid sentinel lymph node biopsy. Patients without HER2 expression are at a greater likelihood of false-negative results; therefore, the findings of ultrasound that axillary lymph nodes are intact (PAUS-negative results) should be interpreted with caution.

## 1. Introduction

Tumor size and axillary nodal status are the most important prognostic factors for breast cancer in predicting overall and disease-free survival [1]. The risk of developing metastasis mainly depends on tumor biological characteristics [2]. Recently, the influence of biological tumor characteristics on the strategy of adjuvant treatment is increasing, and the value of estimation of axillary lymph node status is decreasing [2].

With emerging breast cancer screening programs, more frequently breast cancer is diagnosed at its early stage, and axillary nodal negative disease accounts for 70% to 85% of cases [3]. Today, all patients undergo a sentinel lymph node biopsy (SLNB) in order to determine axillary nodal status; however, the majority of patients do not need this surgical intervention [4,5,6,7]. Considering that both SLNB and lymphadenectomy (axillary lymph node dissection, ALND) are not treatment procedures, but are performed with the aim of tumor staging, recently much attention has been given to the identification of non-invasive procedures for diagnostic purposes [8]. Preoperative axillary ultrasound (PAUS) is a first-line method to evaluate axillary nodal status [9,10,11]. Currently, there are two ongoing clinical studies (SOUND and INSEMA) that aim at evaluating differences in overall survival, disease-free survival, and quality of life in women with breast cancer by dividing them into two groups: women who underwent SLNB and women who had clinically and sonographically tumor-free axillary lymph nodes (c/sN0) and did not undergo SLNB [12,13]. It is assumed that preoperative ultrasound examination allows the identification of patients with clinically important axillary node lesions (metastases in ≥3 lymph nodes), and if axillary lymph nodes appear to have normal appearance on ultrasound (PAUS-negative), SLNB could be avoided. 

The aim of our study was to determine the prognostic value of PAUS while evaluating that axillary lymph nodes are tumor free and to assess the accuracy of PAUS while identifying a heavy nodal disease burden (≥3 metastases in axillary lymph nodes). An additional objective was to determine a false-negative rate in predicting any nodal positivity and a heavy nodal disease burden as well as to examine what clinicopathological factors were associated with this.

## 2. Materials and Methods

This prospective study enrolled women with newly diagnosed breast cancer, who were treated in the Breast Surgery Unit, Clinic of Surgery, Hospital of Lithuanian University of Health Sciences Kauno Klinikos, between May 1, 2016, and May 31, 2018. The study was approved by Kaunas Regional Biomedical Research Ethics Committee (permission No. BE-2-25, Approved on July 18, 2016). 

The exclusion criteria were as follows: (1) patient’s refusal to participate; (2) neoadjuvant chemotherapy; and (3) and recurrent breast cancer. Of the 328 patients examined, 227 women with pT1 breast cancer were enrolled in this study. Bilateral breast cancer was analyzed as two separate cases. 

One day before surgery, sentinel lymph nodes were marked by a 0.2 mL periareolar injection of 50 MBq of 99 mTC-Nanocoll. On the skin, the location of the sentinel lymph node (SLN) was detected using a 2010 Philips BrightView Gamma Camera.

**Figure 1:**
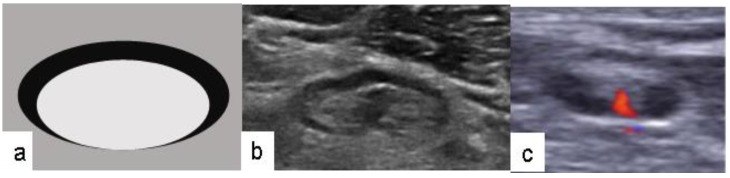
Lymph node with normal appearance: (**a**) schematic drawing; (**b**) signs of preoperative axillary ultrasound-negative lymph node (oval shape, smooth cortex, unchanged, clearly visible fat gate); (**c**) central vascularity.

**Figure 2:**
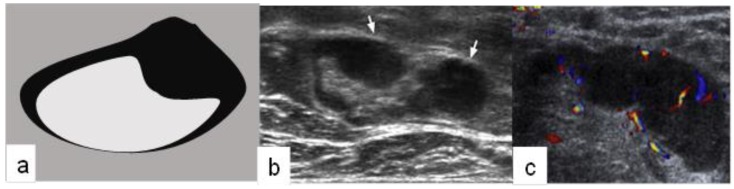
Suspicious lymph node: (**a**) schematic drawing; (**b**) signs of PAUS-positive lymph node (oval or rounded shape, local thickening of the cortex, dislocated gate); (**c**) mixed or peripheral vascularity.

On the day of surgery, all patients underwent repeated PAUS of the targeted areas of SLNs. PAUS was performed using an Acuson S2000 ultrasound unit with a 14 MHz linear Siemens transducer. The PAUS examination assessed whether lymph nodes were normally appearing (PAUS-negative) or were suspicious (PAUS-positive). The signs of PAUS-negative axillary lymph node were as follows: (1) oval shape; (2) smooth cortex <3 mm; (3) unchanged, clearly visible fat gate; and (4) central vascularity (Figure 1) [1,14]. The signs of PAUS-positive axillary lymph node were as follows: (1) oval or rounded shape; (2) local thickening of the cortex >3 mm; (3) dislocated gate; and (4) mixed or peripheral vascularity (Figure 2) [3,15,16].

During surgery, the location of the SLN was detected with a Crystal Probe automatic hand-held gamma detector. Surgically removed radioactive lymph nodes were subjected to pathological examination (hematoxylin and eosin staining and immunohistochemistry). According to the recommendations of the ACOSOG Z0011 study, after SLNB, if metastases were found only in one or two SLNs, axillary lymph node dissection (ALND) was not performed; if metastases were found in three and more sentinel lymph nodes (heavy nodal disease burden), ALND was performed [17]. If an SLN was not found, ALND was also performed. The following histopathological data were analyzed: histological tumor type, presence of non-invasive component (ca in situ), tumor size (pT), tumor grade (G), lymphovascular invasion (LV), presence of estrogen (ER) or progesterone (PR) receptors, human epidermal growth factor (HER2) status, and lymph node (N) status. Nodal metastasis was defined as the presence of macrometastasis (>2.0 mm) (node-positive); lymph nodes without tumor cells or with only isolated tumor cells (<0.2 mm) or with micrometastasis (0.2–2 mm) were considered negative (node-negative) [13,18,19,20]. The nodal disease burden was categorized as a limited nodal tumor burden (without or with 1 or 2 tumor-involved lymph nodes) and a heavy nodal tumor burden (≥3 tumor-involved lymph nodes). PAUS data were compared with the results of histological examination.

### Statistical Analysis

Statistical analysis was performed using IBM SPSS Statistics 25 package (IBM Corp. Released 2017. IBM SPSS Statistics for Windows, Version 25.0. Armonk, NY, USA: IBM Corp). Descriptive statistics methods were used to systemize the study data. The accuracy, sensitivity, specificity, positive-predictive value (PPV), and negative-predictive value (NPV) of the ultrasound examination were evaluated, and the estimates of these characteristics were given along with their 95% confidence intervals (CIs). The chi-square test for independence (homogeneity) was used to compare the effects of clinicopathological factors and the correlation of outcomes from PAUS and SLNB pathology. The Student *t* test was used to compare continuous data with normal distribution. The observed differences and associations were considered statistically significant if *p* < 0.05. 

## 3. Results

The study analyzed 227 women aged 32 to 85 years, with a mean age of 59 years (SD, 11.83). The patients were divided into three groups according to age: ≤50 years (n = 55, 24.2%), 50–70 years (n = 125, 55.1%), and >70 years (n = 47, 20.7%). Women with pT1c breast cancer accounted for the largest proportion (68.3%). Unifocal tumors were more prevalent (n = 172, 75.8%); tumor multifocality was documented in 55 cases (24.2%). Infiltrative ductal carcinoma was the most common histological type (74.0%). The non-invasive component was found in 33.0% of cases. The majority (79.3%) of the tumors were of medium differentiation grade (G2). Lymphovascular invasion (LV1) was recorded in 60.8% of the cases, and 39.2% of the cases did not show lymphovascular invasion (LV0). Hormone-sensitive tumors were more common (ER(+) 88.1% and PR(+) 78.9%). Nearly two-thirds (63.0%) of the tumors were HER2(0) and 37%, HER2(1). Patients’ demographic characteristics and tumor clinicopathological data by node-negative and node-positive groups are shown in Table 1. 

**Figure 3:**
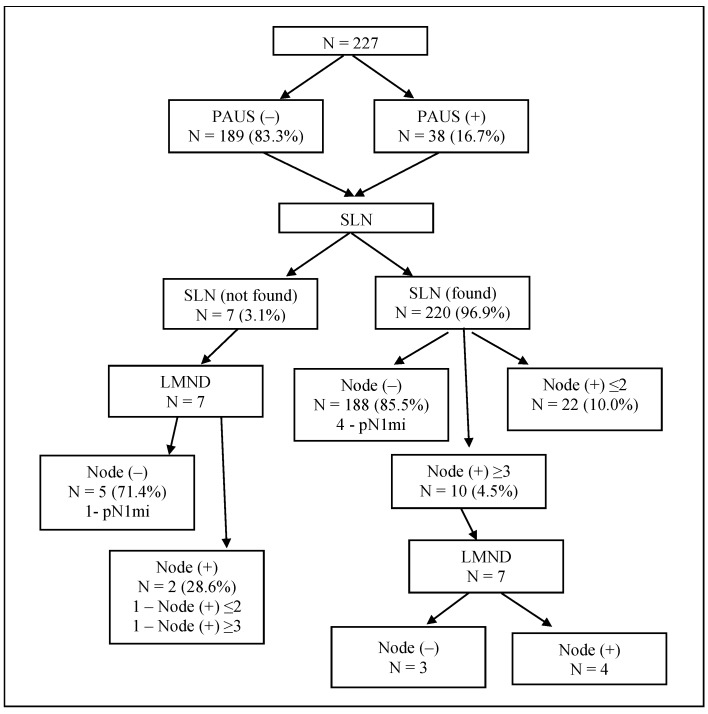
Flowchart of involvement of axillary lymph nodes in 227 patients. PAUS, preoperative axillary ultrasound; SLN, sentinel lymph node; LMND, lymphadenectomy.

**Figure 4:**
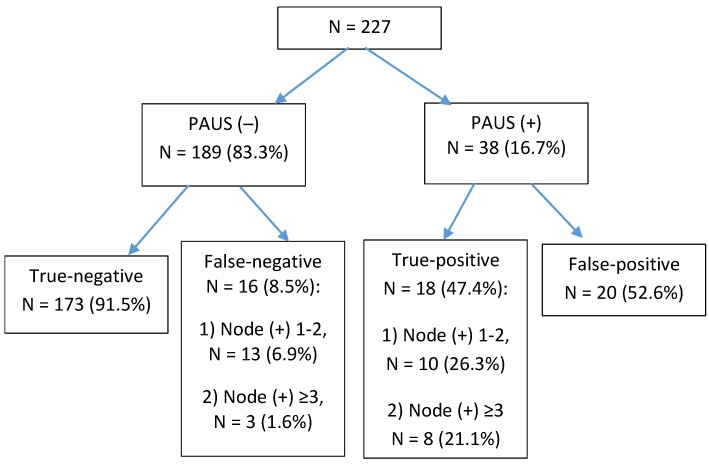
Flowchart of preoperative axillary ultrasound and histological examination findings in 227 patients. PAUS, preoperative axillary ultrasound.

On ultrasound, suspicious lymph nodes (PAUS-positive) were identified in 38 cases (16.7%), and in the remaining 189 cases (83.3%), axillary lymph nodes had normal appearance (PAUS-negative). During surgery, the SLN was found in 96.9% of the cases. Of these, additional lymphadenectomy (LMND) was performed in 3.2% of the cases because 3 SLNs were found and they all were node-positive. In 1.3% of the cases, 4–6 SLNs were excised; metastases were found in 3 lymph nodes, and no ALND was performed. The SLN was not found in 3.1% of the cases, and these patients also underwent LMND (Figure 3). Of the all examined patients, 193 patients (85.0%) were node-negative (5 cases of pN1mi); metastases were present in 34 cases (15.0%). Metastases in ≤2 lymph nodes were found in 23 cases (10.1%); a heavy nodal burden (≥3 positive nodes) was documented in 11 cases (4.8%) (Figure 3). The results of ultrasound and histological examination of axillary lymph nodes are displayed in Figure 4.

The specificity, accuracy, and NPV of ultrasound examination were found to be high, i.e., 89.6%, 84.1%, and 91.5%, respectively; while the sensitivity was moderate being 52.9%. In ≥ 3 node-positive cases, the sensitivity of PAUS increased to 72.7%; accuracy to 88.7%; and NPV to 98.3%. In total, false-negative results were recorded in 8.5% (n = 16) of the cases; in the PAUS-negative group, false-negative results were found only in 1.6% (n = 3) of the cases. Diagnostic discrimination of PAUS is shown in Table 2.

Histopathological tumor data and true-negative and false-negative results were analyzed. The results of ultrasound and pathological examination significantly differed between patients without lymphovascular invasion (LV0) and those who had lymphovascular invasion (LV1). Paired comparison showed the true-negative rate was statistically significantly different between the LV0 and LV1 groups (91% vs. 66.7%, *p* < 0.05). False-negative and true-positive results were documented only in the LV1 group, and the false-positive rate was the same in both groups (9% vs. 8.7% in the LV0 and LV1 groups, respectively; *p* > 0.05). In the LV0 group (n = 89), all patients were node-negative (91% true-negative and 9% false-positive).

Moreover, the results of ultrasound and pathological examination were significantly different between patients without the expression of HER2 (HER2(0)) and those with weakly or strongly expressed HER2 (HER2(1)) (*p* = 0.024). Paired comparisons showed that the false-negative rate was statistically significantly different between the HER2(0) and HER2(1) groups (10.5% vs. 1.2%, *p* < 0.05). True-negative, true-positive, and false-positive rates were similar in both the HER2(0) and HER2(1) groups (74.8% vs. 78.6%, 8.4% vs. 7.1%, 6.3% vs. 13.1%, respectively; *p* > 0.05). 

Evaluation of other characteristics such as tumor size, multifocality, histological type, presence of non-invasive component, differentiation grade, estrogen/progestin receptor status, and patients’ age showed patients’ groups to be homogenous.

## 4. Discussion

Early stage breast cancer is defined as disease confined to the breast with or without regional lymph node involvement and the absence of distant metastatic disease [21]. The role of SLNB is controversial. According to the literature, approximately 60% to 85% of early breast cancer cases are node negative, thus would not benefit from SLNB [1,2,5,22,23,24,25,26]. In our study, 85.0% of the cases were node-negative, and axillary surgical interventions were unnecessary. Metastatic lymph nodes were detected only in 15.0% of the cases, and it might be caused by the fact that we included women with tumors of small size (pT1) to this study. Several studies have reported that the frequency of axillary lymph node metastases depends on tumor size, and it varies between 10% and 26% in pT1 tumors [25,26,27,28].

The SLN was found in 96.9% of the cases; of them, additional LMND was performed in 3.2% of the cases. In additionally removed lymph nodes, metastases were found in 57.1% of the cases, while in the remaining cases, additionally removed nodes were node-negative. According to other studies in the literature, ALND followed by positive results for metastasis at SLNB showed the absence of metastasis of 38% to 67% in the remaining lymph nodes [29,30,31].

Based on literature data, surgeons identify an SLN in 79% to 98% of cases, and in 2–21% of cases, an SLN is not detected [4,32,33,34]. Such varying results depend on different surgeons’ experience [33,34]. In our study, the SLN was not found in 3.1% of the cases. These patients also underwent LMND, and 71.4% of the cases were node-negative, thus did not benefit from surgical intervention. 

One of the objectives of this study was to determine the NPV of PAUS. The reported NPV of PAUS for predicting negative nodal status varies widely in the literature, from 68 to 98% [15,16,23,35,36,37,38]. In our study, the NPV was very high (91.5%, 95% CI, 86.5% to 96.6%), and it could be due to small tumor size (pT1), therefore, leading to a lower metastasis incidence. Accordingly, negative axillary ultrasound excluded axillary metastatic disease in 91.5% of the patients. These data suggest that negative axillary ultrasound may replace SLNB in the majority of cases when the tumor size is small. In our study, the accuracy of the ultrasound examination was also quite high (84.1%). In other studies, it varied from 66% to 90.2% [2,8,10,11,35,39].

Most previous studies have focused on PAUS ability to predict any nodal involvement [2,7,8,36,39,40]. Some studies examined ultrasound ability to discriminate between minimal and heavy axillary metastatic burden. In all these studies, the NPV and the accuracy of the PAUS increased to 86–98.4% and 81.2–98.5% in cases with a heavy nodal burden [10,11,16,35]. In the current study, the NPV and the accuracy of PAUS in detecting a heavy nodal burden increased to 98.3% and to 88.7%, respectively. 

Our additional objective was to examine patients’ and tumor characteristics associated with false-negative results of PAUS. Our study showed that in the PAUS-negative group, a heavy nodal burden was found only in 1.6% of the cases (false-negative rate). Patients’ and tumor characteristics associated with false-negative axillary ultrasound in several studies are shown in Table 3. The study by Jackson et al. showed a false-negative rate of PAUS being 4% (95% CI, 2–6%) for detecting ≥3 nodal metastasis [10]. In this study, cases with a different tumor size (pT1, pT2, pT3, pT4) were included. The false-negative rate in the pT2-pT4 group was 8.2%, and in the pT1 group, it was 1.7%, being very similar to the rate in our study [10]. Several studies have demonstrated that larger tumor sizes were more often associated with false-negative results of ultrasound [2,11,41,42,43]. These results show that smaller tumors are generally associated with a lower likelihood of nodal positivity, so a lower false-negative rate could be expected with lower pathologic T size. 

Two studies identified a relationship between false-negative PAUS and histological tumor type. In the false-negative PAUS group, lobular carcinoma was more frequently diagnosed than other types of carcinoma [10,42]. In our study, we did not find any association between false-negative PAUS rate and tumor histological type. It might be caused by a small number of patients with lobular carcinoma in our study.

Some previous studies have reported associations between higher tumor grade (G3) and an increased likelihood of false-negative PAUS results [2,11]. However, we did not find any difference in the false-negative rate depending on tumor grade.

The study by Meretoja et al. demonstrated that false-negative axillary ultrasound was more frequently documented in patients who had multifocal tumor versus patients who had unifocal tumor [43]. In our study, there was no significant difference in the false-negative PAUS rate comparing patients with multifocal and unifocal disease.

Several previous studies have shown an association between lymphovascular invasion in the breast and false-negative results of PAUS [2,11,41,42,43]. In the study by Johnson et al., the false-negative group was more likely to have lymphovascular invasion in the breast than the true-negative one (5% vs. 31%, *p* < 0.001) [42]. The study by Nwaogu et al. showed that lymphovascular invasion was also more likely to occur in the false-negative group compared with the true-negative group (44% vs. 8%, *p* < 0.0001) [41]. The study by Artmoniene et al. demonstrated that in cases with LV1, the false-negative rate of PAUS was greater (V1 54.6% and L1 98.3%), and the true-negative rate of PAUS was lower (V1 23.5% and L1 29.6%) (*p* < 0.0001) [11].

We found significant differences in PAUS results between patients without lymphovascular invasion (LV0) and with lymphovascular invasion (LV1). The true-negative rate of PAUS was significantly different between the LV0 and LV1 groups. False-negative results of PAUS were recorded only in the LV1 group. Moreover, we showed that in the LV0 group (n = 89), all patients were node-negative (91% of true-negative and 9% of false-positive); therefore, we can rely on PAUS examination that axillary lymph nodes are intact.

Our study also revealed significant differences in PAUS results between patients without and with HER2 expression. There was a significant difference in the false-negative rate of PAUS comparing the HER2(0) and HER2(1) groups (10.5% and 1.2%, respectively; *p* < 0.05). In the study by Stachs et al., the false-negative PAUS rate was also more frequently found in the HER2(0) than the HER2(1) group (56% vs. 18.8%, *p* = 0.007). Therefore, the assessment of axillary ultrasound may be less accurate in this subset, and a negative result should be interpreted with caution. Other features had no impact on the false-negative rate of PAUS. 

Our study had some limitations to be mentioned. First, the study population included a relatively small number of patients from a single institution. Second, in this study, ultrasound evaluation was performed by one radiologist who had 10-year work experience. In the future, it would be interesting to include more institutions and more radiologists with different work experience and then to compare the obtained results. PAUS is a valuable and cost-effective imaging tool that could replace SLNB in majority of cases when the tumor size is small.

## 5. Conclusions

Negative axillary ultrasound excluded axillary metastatic disease in 91.5% of the patients; therefore, sentinel lymph node biopsy itself represented surgical over-treatment in these patients. PAUS had an accuracy of 88.7% in detecting a heavy nodal disease burden. In the LV0 group, PAUS results were found to be reliable and SLNB could be avoided. The probability of false-negative PAUS rate was higher in patients without HER2 expression; therefore, negative PAUS findings in patients with HER2(0) should be interpreted cautiously.

## Figures and Tables

**Table 1:** Patients’ demographic and tumor clinicopathological characteristics by node-negative and node-positive groups.

Parameter	Total (n = 227)	Node-Negative (n = 193)	Node-Positive (n = 34)	*p* Value
Age, mean (SD), years	59.55 (11.83)	59.88 (11.93)	57.65 (11.21)	0.311
Age categories: ≤50 years 50–70 years >70 years	55 125 47	45 (81.8) 106 (84.8) 42 (89.4)	10 (18.2) 19 (15.2) 5 (10.6)	0.565
Findings on PAUS, n (%): Negative Positive	189 38	173 (91.5) 20 (52.6)	16 (8.5) 18 (47.4)	<0.001
Number of focus, n (%): Unifocal Multifocal	172 55	146 (84.9) 47 (85.5)	26 (15.1) 8 (14.5)	0.556
Histological subtype, n (%): Invasive ductal Invasive lobular Other	168 19 40	143 (85.1) 14 (73.7) 36 (90.0)	25 (14.9) 5 (26.3) 4 (10.0)	0.260
Presence of non-invasive component, n (%): ca in situ (+) ca in situ (–)	75 152	64 (85.3) 129 (84.9)	11 (14.7) 23 (15.1)	0.548
Tumor size, n (%): pT1a pT1b pT1c	27 45 155	26 (96.3) 40 (88.9) 127 (81.9)	1 (3.7) 5 (11.1) 28 (18.1)	0.112
Tumor grade, n (%): G1 G2 G3	23 180 24	22 (95.7) 152 (84.4) 19 (79.2)	1 (4.3) 28 (15.6) 5 (20.8)	0.255
Lymphovascular invasion, n (%): LV0 LV1	89 138	89 (100.0) 104 (75.4)	0 (0.0) 34 (24.6)	<0.001
HR status, n (%): ER negative ER positive	27 200	25 (92.6) 168 (84.0)	2 (7.4) 32 (16.0)	0.191
PR negative PR positive	48 179	41 (85.4) 152 (84.9)	7 (14.6) 27 (15.1)	0.568
HER2 status, n (%): HER2(0) HER2(1)	143 84	116 (81.1) 77 (91.7)	27 (18.9) 7 (8.3)	0.022

Categorical data were compared with the chi-square test; continuous data, with the Student *t* test. PAUS, preoperative axillary ultrasound; LV, lymphovascular invasion; HR, hormone receptor; ER, estrogen receptor; PR, progesterone receptor; HER2, human epidermal growth factor receptor 2.

**Table 2:** Diagnostic discrimination of PAUS in patients with a limited nodal burden and a heavy nodal burden.

PAUS	Prevalence% (95% CI)	Accuracy % (95% CI)	Sensitivity % (95% CI)	Specificity % (95% CI)	Positive Predictive Value % (95% CI)	Negative Predictive Value % (95% CI)
Total	15.0 (9.1–20.9)	84.1 (78.1–90.2)	52.9 (31.5–74.3)	89.6 (84.2–95.1)	47.4 (27.1–67.6)	91.5 (86.5–96.6)
≥3 node+	5.4 (1.4–9.3)	88.7 (83.2–94.3)	72.7 (39.2–106.3)	89.6 (84.2–95.1)	28.6 (7.2–49.9)	98.3 (95.9–100.7)

**Table 3:** Data of several studies: associations between clinicopathological factors and false-negative rate of PAUS.

Author	PAUS-Negative	False-Negative Rate	Feature, *p*-Value
Johnson et al., 2011	N = 155	29% (overall)	Size of tumor (*p* = 0.02), Lymphovascular invasion (*p* < 0.001), Histological type of tumor (*p* < 0.001)
Stachs et al., 2013	N = 378	18.5% (overall)	Tumor size (*p* < 0.001), tumor grade (*p* = 0.005), lymphovascular invasion (*p* < 0.001), ER status (*p* = 0.024), PR status (*p* = 0.014), HER2 status (*p* = 0.007) Ki-67 proliferation index (*p* < 0.001)
Meretoja et al., 2014	–	–	Tumor size (*p* < 0.001), multifocality (*p* < 0.001), lymphovascular invasion (*p* < 0.001), palpability of the primary tumor (*p* < 0.001)
Nwaogu et al., 2015	N = 118	21% (overall)	Tumor size (*p* < 0.01), lymphovascular invasion (*p* < 0.0001)
Jackson et al., 2015	N = 400	14% (overall) 4% (for detecting ≥3 nodal metastasis)	Tumor size (*p* = 0.005), histological tumor type (*p* = 0.004)
Artmoniene et al., 2019	N = 581	18.7%	Tumor size (*p* < 0.0001), tumor grade (*p* = 0.034), lymphovascular invasion (*p* < 0.0001), patient age (*p* = 0.006)
Current study	N = 189	8.5% (overall) 1.6% (for detecting ≥3 nodal metastasis)	Lymphovascular invasion (*p* < 0.0001)
